# Evidence for an association between migraine and the hypocretin receptor 1 gene

**DOI:** 10.1007/s10194-011-0314-8

**Published:** 2011-02-23

**Authors:** Innocenzo Rainero, Elisa Rubino, Salvatore Gallone, Pierpaola Fenoglio, Luigi Rocco Picci, Laura Giobbe, Luca Ostacoli, Lorenzo Pinessi

**Affiliations:** 1 Neurology II, Department of Neuroscience, University of Torino, Via Cherasco 15, 10126 Turin, Italy; 2 Mental Health Department, San Luigi Gonzaga Hospital, University of Turin, Orbassano, Italy

**Keywords:** Migraine without aura, Migraine with aura, Hypocretin receptor 1, Hypocretins, Genetic association studies

## Abstract

The aim of our study was to investigate whether genetic variants in the hypocretin receptor 1 (*HCRTR1*) gene could modify the occurrence and the clinical features of migraine. Using a case–control strategy we genotyped 384 migraine patients and 259 controls for three SNPs in the *HCRTR1* gene. Genotypic and allelic frequencies of the rs2271933 non-synonymous polymorphism resulted different (*χ* ^2^ = 9.872, *p* = 0.007; *χ* ^2^ = 8.108, *p* = 0.004) between migraineurs and controls. The carriage of the A allele was associated with an increased migraine risk (OR 1.42, 95% CI 1.11–1.81). When we divided the migraine patients into different subgroups, the difference reached the level of statistical significance only in migraine without aura. The different genotypes had no significant effect on the examined clinical characteristics of the disease. In conclusion, our data supports the hypothesis that the *HCRTR1* gene could represent a genetic susceptibility factor for migraine without aura and suggests that the hypocretin system may have a role in the pathophysiology of migraine.

## Introduction

Migraine is a chronic neurovascular disease characterized by recurrent headache attacks associated with autonomic, gastrointestinal and focal neurological symptoms [1]. Migraine is a public health problem of great impact on both the patient and society. The prevalence studies showed that migraine affects 15–25% of women and 6–8% of men [2]. Migraine is rated as one of the most disabling chronic disorders by the World Health Organization. The annual cost of migraine-related loss in productivity is enormous and it has been estimated to be the most costly neurological disorder in Europe [3].

Migraine is a complex disorder that shows a strong (up to 50%) genetic component with a probable multifactorial inheritance [4]. Mutations in three genes encoding neural ion channels, CACNA1A, ATP1A2, and SCN1A, have been described in patients with familial hemiplegic migraine (FHM), a rare monogenic, autosomal dominant form of migraine with aura [5]. However, the success of FHM, regarding discovery of genetic defects associated with the disease, remains elusive in the common forms of migraine, and causative genes have not been identified yet [6].

Recently a new neurotransmitter system, the hypocretin orexin system, has been implicated in neurological diseases. This system consists of two neuropeptides transmitters, hypocretin-1 and -2 (also termed orexin-A and -B), that are encoded by a unique precursor, preprohypocretin, and two G-protein coupled receptors (HCRTR1 and HCRTR2), that act via PKC to phosphorilate voltage-activated calcium channels [7, 8]. HCRTR1 has a preferential affinity for hypocretin-1, 50 times more than hypocretin-2, while HCRTR2 has comparable affinities for the two peptides [9].

Hypocretins are produced by a small number of neurons located in the lateral hypothalamic area and in the posterior hypothalamus [10]. The hypothalamic hypocretin neurons may be divided into at least two different populations, a lateral one playing a role in food intake and addiction [11] and a more medial one involved, in particular, in stress and arousal [12]. Hypocretin neurons project to wide targets, as cerebral cortex, olfactory bulb, hippocampus, amygdala, hypothalamus itself, midbrain, spinal cord, and brainstem; in turn, they receive excitatory or inhibitory inputs from the same areas [10]. Hypocretin systems are also involved in circadian pathway, hormones secretion, and in emotional and autonomic processes related to stress as well [13]. They are, therefore, crucial in the regulation of motivational and adaptative behavior to metabolic and environmental stimuli.

Several functions regulated by hypocretins are significantly impaired in patients with migraine and this could be relevant to the pathophysiology of the disease. In particular, premonitory symptoms preceding a migraine attack, such as fatigue, yawning, excessive sleepiness and craving for certain food, indicate an involvement of different hypothalamic nuclei that might be related to the hypocretin functions.

An alteration in orexin signaling makes an attractive candidate to explain several of the symptoms observed in patients with primary headaches. Our research group has previously reported that the 1246G/A polymorphism of the hypocretin receptor 2 (*HCRTR2*) gene is significantly associated with cluster headache [14]. This association was confirmed by a large study performed in Germany, showing that homozygous carriers of the G allele had a twofold increase in risk for the disease [15], but was not replicated in a complex dataset of CH patients of Danish, Swedish, and British origin [16]. Conversely, analyzing the same polymorphism in migraine, no statistical difference was found between cases and controls [17] and the same results were obtained by an independent group [18]. Thereby, in order to deepen the analysis of hypocretin system in migraine pathogenesis, we hypothesized that *HCRTR1* gene polymorphisms would modify the occurrence and the clinical features of migraine patients. To test this hypothesis, we performed a case–control association study in a cohort of Italian migraine patients and in healthy controls.

## Methods

### Patients and controls

A total of 384 consecutive migraine patients (110 males, 274 females; mean age ± SD = 41.2 ± 13.2, mean age at onset of disease ± SD = 18.5 ± 10.0 years), attending the Headache Center of the University of Torino (Italy), and the Psychiatric Clinic (St. Louis Hospital) of the University of Torino, were involved in the case–control association study. The diagnoses of Migraine without aura (MO) (330 patients) and Migraine with aura (MA) (54 patients) were made according to the International Classification of Headache Disorders (IHCD-II) criteria [19]. A standardized record of all the clinical and psychological characteristics of migraine, suitable for computer analysis, was obtained. A group of 259 sex, age and geographically matched healthy subjects (81 males, 178 females, mean age ± SD = 41.9 ± 12.9 years) were used as controls. The controls were healthy blood donors and were screened by a neurologist specialized in headaches in order to exclude migraine or chronic tension-type headache. Written informed consent was obtained from all participants and the study was approved by the Hospital Ethics Committee.

### Genetic analysis

Genomic DNA was extracted using the QIAamp® Mini Kit (Qiagen S.p.A., Italy). We genotyped cases and controls for three bi-allelic polymorphisms (SNP1 rs10914456, SNP2 rs4949449, and a non-synonymous SNP3 rs2271933 (I408 V) of the *HCRTR1* gene, selected from SNPs database of NCBI (http://www.ncbi.nlm.nih.gov/). These polymorphisms have been shown to be polymorphic in Western populations. PCR reactions were performed according to standard conditions. Supplementary data are available on request.

### Statistics

*χ* ^2^ test was used in verifying Hardy–Weinberg equilibrium. Statistical analyses were performed using Genepop—version 4.0 (http://wbiomed.curtin.edu.au/genepop), SigmaStat—version 3.1 (Jandel Corp., 1994, San Rafael, CA, USA), SVS 7 (http://www.goldenhelix.com) and SPSS version 17. SNPs were assessed for both genotypic and allelic association as well as the Armitage trend test. ANOVA followed by Bonferroni correction for multiple comparisons were used to compare the clinical characteristics between cases and controls. Haploview program version 4.1 (http://www.broad.mit.edu/mpg/haploview/) was used for haplotype analysis and for pairwise linkage disequilibrium, D′ and *r* ^2^. Genetic Power Calculation (http://statgen.iop.kcl.ac.uk/gpc) was used to calculate the power of the association study. According to the recent guidelines for genetic association studies in neurological disorders, the level of statistical significance was taken at *p* < 0.01 [20], whereas for other comparisons the level of significance was taken at *p* < 0.05.

## Results

### Association between *HCRTR1* gene and migraine

Table 1 shows the genotype (GF) and the allele frequencies (AF) of the three polymorphisms examined in the *HCRTR1* gene (rs10914456, rs4949449, and rs2271933) and the comparison between healthy controls and migraine patients. Hardy–Weinberg equilibrium was verified for all populations (data on request). No significant differences were found in the distribution of either genotypic or allelic frequencies between cases and controls in *HCRTR1* rs10914456 and rs4949449.

**Table 1:** Genotype and Allele Frequencies of the *HCRTR1* polymorphisms in migraine patients and controls

	GF			AF	
HCRTR1 SNP1 rs10914456	C/C (%)	C/T (%)	T/T (%)	C (%)	T (%)
Controls	108 (41.7)	122 (47.1)	29 (11.2)	338 (65.3)	180 (34.7)
Migraine patients	146 (38.1)	188 (49.0)	50 (13.0)	480 (62.5)	288 (37.5)
HCRTR1 SNP2 rs4949449	G/G (%)	G/T (%)	T/T (%)	G (%)	T (%)
Controls	104 (40.2)	129 (49.8)	26 (10.0)	337 (60.6)	181 (39.4)
Migraine patients	147 (38.3)	190 (49.5)	47 (12.2)	484 (63.0)	284 (37.0)
HCRTR1 SNP3 rs2271933	G/G (%)	G/A (%)	A/A (%)	G (%)	A (%)
Controls	125 (53.3)	108 (41.7)^a^	26 (10.0)^a^	358 (69.1)	160 (30.9)^b^
Migraine patients	138 (36.0)	194 (50.5)	52 (13.5)	470 (61.2)	298 (38.8)

*p* values were calculated by the *χ* ^2^ test from 3×2 (genotype) or 2×2 (allele) contingency tables

*GF* genotypic frequency, *AF* allelic frequency

^a^ GF *χ* ^2^ = 9.872, *p* = 0.007, power = 0.819

^b^ AF *χ* ^2^ = 8.108, *p* = 0.004, power = 0.828

Conversely, analyzing the rs2271933 (1222G>A) non synonymous polymorphism, a statistical difference was found in the distribution of GF and AF between cases and controls. The allelic frequency for 1222G was 0.69 in controls and 0.61 in migraine patients, AF for 1222A was 0.31 in controls and 0.39 in migraineurs (*χ* ^2^ = 8.108, *p* = 0.004, power = 0.828). The carriage of the A allele was associated with a significantly increased disease risk (OR 1.42, 95% CI 1.11–1.81). The distribution of genotypic frequencies between cases and controls resulted significantly different (*χ* ^2^ = 9.872, *p* = 0.007, power = 0.819) as well. To assess the dosage effect of possessing zero, one or two copies of the A-risk allele, according to an additive model, the Armitage test for linear trend in proportion was performed on the genotype frequency data. However, the obtained *p* values were not significant after Bonferroni correction. When analyzing the dominant model, the comparison between GA+AA vs. GG showed a difference between cases and controls (*χ* ^2^ = 9.217, *p* = 0.002, power = 0.877), with an increased risk of migraine in GA+AA carriers compared with GG carriers (OR = 1.66; 95% CI = 1.19 < OR < 2.32), according to a dominant model (Table 2). The permutation test of one million times generated a total *χ* ^2^ of 8.518 (*p* = 0.0057) in order to observe a significant difference in rs2271933 analysis. When analyzing the gender effect, the association according to the dominant model was confirmed only in female patients (*p* = 0.003, OR = 1.80; 95% CI = 1.22 < OR < 2.65) and not in males, probably due to the low-number of this subgroup. The present study had a power of 0.82 to detect a significant association, assuming a prevalence of migraine = 0.12, a high-risk allele frequency = 0.3, a genotypic relative-risk GA = 1.5, and a genotypic-risk relative-risk AA = 2.

**Table 2:** Genotypic, allelic, and haplotypic comparisons in *HCRTR1* gene I408V polymorphism between migraine patients and controls

HCRTR1 SNP3 I408V (rs2271933)	Chi	*p*	OR	95% CI
Alleles				
A vs. G	*χ* ^2^ = 8.108	*p* = 0.004	1.42	1.11 < OR < 1.81
Genotypes				
AA vs. GG	*χ* ^2^ = 4.355	*p* = 0.037	1.81	1.03 < OR < 3.19
GA vs. GG	*χ* ^2^ = 7.556	*p* = 0.006	1.42	1.11 < OR < 1.81
AA vs. GA	*χ* ^2^ = 0.071	*p* = 0.789	1.11	0.64 < OR < 1.95
GA+AA vs. GG (dominant model)	*χ* ^2^ = 9.217	*p* = 0.002	1.66	1.19 < OR < 2.32
AA vs. GG + GA (recessive model)	*χ* ^2^ = 1.467	*p* = 0.226	1.40	0.83 < OR < 2.30
Haplotypes				
TTA	*χ* ^2^ = 6.151	*p* = 0.013	1.37	1.07 < OR < 1.74
TTG	*χ* ^2^ = 25.537	*p* = < 0.001	0.10	0.03 < OR < 0.30

When we divided the migraine patients into two different subgroups (migraine with aura—MA—and migraine without aura—MO), according to IHCD-II criteria, the difference reached the level of statistical significance only in MO subgroup. The different genotypes had no significant effect on the examined migraine clinical features (nausea, vomiting, phono and photophobia, pulsating pain, unilateral pain, age at onset of the disease, and duration of the disease and frequency of migraine attacks) (Table 3).

**Table 3:** Clinical characteristics of migraine patients according to HCRTR1 I408V different genotypes

	G/G	G/A	A/A	*p* value
Number	138	194	52	
Age at onset of the disease ± SD (years)	23.22 ± 14.08	21.10 ± 11.67	23.04 ± 12.32	*p* = 0.278
Duration of the disease ± SD (years)	19.93 ± 13.60	22.69 ± 13.37	21.39 ± 12.85	*p* = 0.181
Frequency of migraine attacks (attacks for year)	51.00 ± 61.69	47.08 ± 57.44	41.11 ± 62.64	*p* = 0.584
Nausea	97	142	39	*p* = 0.847
Vomiting	54	83	14	*p* = 0.292
Phono or photophobia	110	164	45	*p* = 0.514
Unilateral pain	31	131	82	*p* = 0.055
Pulsating pain	96	142	40	*p* = 0.639

## Haplotype block structure analysis of *HCRTR1* gene

Multilocus haplotypes are usually more informative than any single marker, so we performed a haplotype analysis of *HCRTR1* gene. Pairwise analysis showed that the *HCRTR1* SNPs are in high LD: D′ more than 0.91, *r* ^2^ more than 0.7 and LOD more than 55 and was found for the three nearby SNPs. In our Italian population, the latter results evidenced a strong LD in the HCRTR1 region, according to other studies [19], suggesting that *HCRTR1* gene is located on the same LD block. For the *HCRTR1* gene, the analysis identified a total of eight different haplotypes, but only four exceed 1% value (Table 4). The TTG and TTA haplotypes resulted significantly different in cases and controls (*χ* ^2^ = 25.537, *p* < 0.0001; *χ* ^2^ = 6.151, *p* = 0.013). The permutation test (one million times) generated identical values. The carriage of the TTA haplotype was associated with a significantly increased disease-risk (OR 1.37, 95% CI 1.07–1.74) (Table 2). The latter data confirms the differences between cases and controls, underlining in particular the rs2271933 G>A role.

**Table 4:** Haplotypes of the *HCRTR1* polymorphisms in migraine patients and controls

	Controls		Migraineurs	
	*N*	(%)	*N*	(%)
Haplotype				
CGG	325	0.628	450	0.586
TTA	149	0.287	273*	0.356
TTG	26**	0.051	4	0.005
CGA	7	0.012	20	0.025
CTG	4	0.008	5	0.007
TGA	3	0.006	3	0.004
TGG	2	0.004	10	0.013
CTA	2	0.004	3	0.004
	518		768	

* *p* = 0.01

** *p* = < 0.001

## Discussion

The present study of an Italian population provides evidence of a genetic association between a non-synonymous polymorphism (G1222A) in exon 7 of *HCRTR1* gene and migraine. Subject carriers for A allele showed an increased disease risk in comparison with G carriers. Patients with the AA genotype showed an approximately twofold increased risk for migraine compared with carriers of the GG genotype, while subjects with GA genotype had a modest, but significant 1.42-fold risk of developing the disease compared with homozygotes for the G allele. Haplotype analysis confirmed the association. When the patients were divided into different clinical subgroups (migraine with and without aura), the significant difference in gene polymorphism frequencies was found only in migraineurs without aura. Finally, the different HCRTR1 G1222A genotypes did not seem to modify the main clinical features of the disease.

To the best of our knowledge, this is the first study evaluating the association between migraine and the *HCRTR1* gene and additional studies are needed to confirm our findings. Genetic association studies are exposed to several biases, including phenotypic definition of the disease, adequate sample size of patients and controls, selection of the polymorphisms, and population stratification. However, our study had a power of 0.82 to detect a significant association, even if the possibility of undetected bias cannot be excluded. In our study, we found a significant association exclusively in female patients. However, the power to detect a significant association in males was low and these results need to be interpreted with caution. In animal studies, HCRTR1 expression was significantly higher in female hypothalamus with respect to males [20], showing a sexual dimorphism in the expression of hypocretin receptors. When we divided the migraine patients into different subgroups, the difference reached the level of statistical significance only in migraine without aura subgroup. In literature, MO and MA are extensively discussed to be defined as different disease entities [21] with a different genetic background, and our results could further support this view.

The G>A polymorphism of the *HCRTR1* gene leads to an aminoacid substitution of isoleucine at position 408 by valine, which could engender an altered receptor function. Ile408Val mutation is located in the cytoplasmatic tail. It is, therefore, a potential binding site for G proteins, and could alter intracellular signal transduction. However, it is unclear whether this variation may change the function of the protein, modifying its affinity for ligands, its coupling to effectors, its dimerization, or its formation of heterodimers (e.g., with cannabinoid CB1 receptors [22]). This genetic variant was reported to be a benign polymorphism in human narcolepsy [23]. Additionally, an association between the aforementioned polymorphism in polydipsic-hyponatremic schizophrenic patients when compared with non-polydipsic patients was observed, but the study failed to detect a difference in intracellular calcium in mutant cell lines [21].

Etiopathogenetic mechanisms underlying migraine are poorly understood [21]. Many migraineurs experience premonitory autonomic and endocrine symptoms preceding the onset of a migraine attack that may indicate a primary hypothalamic dysfunction [24]. A recent paper highlighted the importance of hypothalamus in migraine pathophysiology [25]. This hypothesis is also supported by an experimental study that demonstrated the existence of a hypothalamic activation in migraine attacks [26] with the positron emission tomography (PET). The interrelation between hypothalamus and interconnected brainstem area could play a key role in migraine physiopathology. In the hypothalamus, HCRTR1 mRNA is densely expressed in the anterior and posterolateral hypothalamus. Outside the hypothalamus, HCRTR1 mRNA are detected in the dorsal raphe nucleus, thalamus, rhombencephalon, periaqueductal gray, hippocampus, spinal cord and dorsal root ganglia and most prominently, in the noradrenergic neurons of the locus coeruleus [27]. The importance of hypocretinergic projections is stressed by their influence on a wide range of many physiological and behavioral processes like as food-intake, pain modulation, sleep-wake cycle and vigilance, reward processing, stress responses, and regulation of autonomic system [13, 28].

Experimental data suggest that the posterior hypothalamus is involved in the modulation of nociceptive processing in humans [29]. The hypothalamic hypocretinergic system projects of many areas involved in pain processing within the CNS and the hypocretins have been suggested to play a role in pain pathway. Stimulation of nociceptive trigeminovascular afferents leads to activation of neurons in the posterior hypothalamus [30]. An intrathecal administration of hypocretin-1 produces analgesic effects in experimental animals [31] and injection of orexin-A reduced mechanical allodynia in a neuropathic pain model [32]. In addition, the administration of a selective non-peptide Hcrt-1 antagonist (SB-334867) reversed the hypocretin-1 analgesic effects, suggesting the presence of a descending orexinergic inhibitory system [33]. Finally, after hypocretin microinjection into the posterior hypothalamus, hypocretin-1 decreased the A- and C-fiber responses to dural and electrical stimulation: this suggests a link between the hypocretinergic system and autonomic changes as well as nociceptive phenomena observed in primary headache disorders [34].

Intriguingly, the recent findings suggested an exciting role for hypocretins in drug addition and reward-seeking. Orexin neurons are stimulated by contextual stimuli associated with cocaine, morphine, ethanol, or food reward [35], indicating that the orexin system may be involved in drug-seeking triggered by associative contexts. Additionally, drug reward, reinstatement of drug seeking and psychomotor sensitization appear to be mediated primarily by HCRTR1, and the blockade of orexin signaling at HCRTR1, via the selective antagonist SB-334867 (SB), has been shown to attenuate cue- and stress-induced reinstatement of cocaine- and ethanol-seeking [36]. About 3–4% of the general population worldwide suffer from chronic daily headache associated with the overuse of headache symptomatic medication making it a significant social problem. A recent research showed significantly higher concentrations of hypocretin-1 in patients with medication overuse headache and in patients with chronic migraine, evidencing a significant correlation with monthly drug intake [37]. Overall, these results support a complex role of hypocretinergic system in motivational state and addictive behaviors to drugs of abuse.

Interestingly, narcoleptic patients exhibit a high-degree of comorbidity with migraine. Narcoleptic patients have low or undetectable hypocretin-1 levels in cerebrospinal fluid (CSF) as well, and show 85–95% reduction in the number of hypocretin neurons in the hypothalamus [38]. Mutations in HCRTR2 that is abundantly expressed in regions highly important for the maintenance of arousal result in narcoleptic symptoms in mice and dogs [39, 40]. Alterations in hypocretin neurotransmission have been found in additional sleep disorders, such as secondary hypersomnia and periodic hypersomnia [41, 42]. At present, new drugs, such as SB649868 and ACT- 078573, which are selective antagonists of hypocretin receptors, are under development for the treatment of neurological disorders. With these exciting premises, the study of the effects of these drugs in patients with primary headaches will provide data of particular interest.

An alternative explanation of our observed data might be related to the linkage disequilibrium with other disease genetic variants in nearby genes, which are responsible for this association. The genetic case–controls association study for complex disorders has the advantage of being powerful to detect loci of small effect size and relatively high-allele frequencies in the population, but it is less sensitive to detect and define loci beyond a narrow distance surrounding the markers compared with linkage studies. At present, genome wide scan studies have identified several loci for migraine susceptibility [23], but none in the 1p33. This chromosomal region could deserve further investigation.

In conclusion, our data supports the hypothesis that the *HCRTR1* gene could represent a genetic susceptibility factor for migraine without aura and that the hypocretin neuronal system may have a role in the pathophysiology of migraine. Additional studies in different population are warranted to confirm and pinpoint our findings.
